# The effect of opioid analgesics, benzodiazepines, gabapentinoids, and opioid agonist treatment on mortality risk among opioid-dependent people.

**DOI:** 10.23889/ijpds.v7i3.1954

**Published:** 2022-08-25

**Authors:** Chrianna Bharat, Natasa Gisev, Sebastiano Barbieri, Tim Dobbins, Sarah Larney, Michael Farrell, Louisa Degenhardt

**Affiliations:** 1 National Drug and Alcohol Research Centre, UNSW Sydney, Sydney, Australia; 2 Centre for Big Data Research in Health, UNSW Sydney, Sydney, Australia; 3 School of Population Health, UNSW Sydney, Sydney, Australia; 4 Université de Montréal and Centre de Recherche du CHUM, Montreal, Canada

## Objectives

As dispensings for benzodiazepines and gabapentinoids have increased in recent years, risk of increased mortality has been identified, particularly when used with opioids. There is limited research examining use of these medicines among people with opioid dependence and whether mortality risk varies according to opioid agonist treatment (OAT) status. This study characterizes patterns of opioid analgesic utilization and concomitant use of benzodiazepines, gabapentinoids and OAT among people with opioid dependence initiating opioid analgesics. It also assesses mortality risk associated with exposure to these medicines.

## Approach

Retrospective cohort study in New South Wales, Australia, including 28,891 people with documented opioid dependence initiating opioid analgesics between July 2003 and December 2018. Linked administrative records provided data on prescription dispensings, sociodemographics, clinical characteristics, OAT and mortality. Generalised estimating equation models estimated incidence rate ratios (IRR) comparing periods in and out of OAT for the number of opioid analgesic dispensings. Periods of concomitant use of opioid analgesics, benzodiazepines, gabapentinoids, and OAT were identified. Cox models assessed associations between concomitant medicines use with mortality risk.

## Results

At the time of opioid analgesic initiation, 43.7% of the cohort were in OAT. The most commonly initiated opioid was codeine (67.8%). In the 90 days prior to the index opioid dispensing, benzodiazepines were more frequently dispensed than gabapentinoids, but rates varied over time. Between 2004 and 2018, benzodiazepine dispensings decreased (41.7% to 21.1%) while gabapentinoid dispensings increased (0.2% to 7.9%). Incidence of opioid analgesic dispensings was higher during periods out of OAT compared to in OAT (5.8 v. 2.3 per person-year; IRR 0.39, 95% CI 0.38, 0.41). Analyses investigating associations between medicine exposure and mortality are ongoing.

## Conclusion

People with opioid dependence had high rates of recent benzodiazepine utilization and current OAT enrollment at the time of opioid analgesic initiation. OAT was associated with a significant reduction in opioid analgesic prescribing.

